# Supplementary material for: Greenhouse gas consequences of the China dual credit policy
Source: Nat Commun. 2020 Oct 15;11:5212. doi: 10.1038/s41467-020-19036-w (PMC7566593; doi:10.1038/s41467-020-19036-w)
Supplement: Supplementary file 1 — Supplementary Information [file 41467_2020_19036_MOESM1_ESM.pdf]

Supplementary Information

Greenhouse Gas Consequences of the China Dual Credit Policy

He et al.

Supplementary Figures

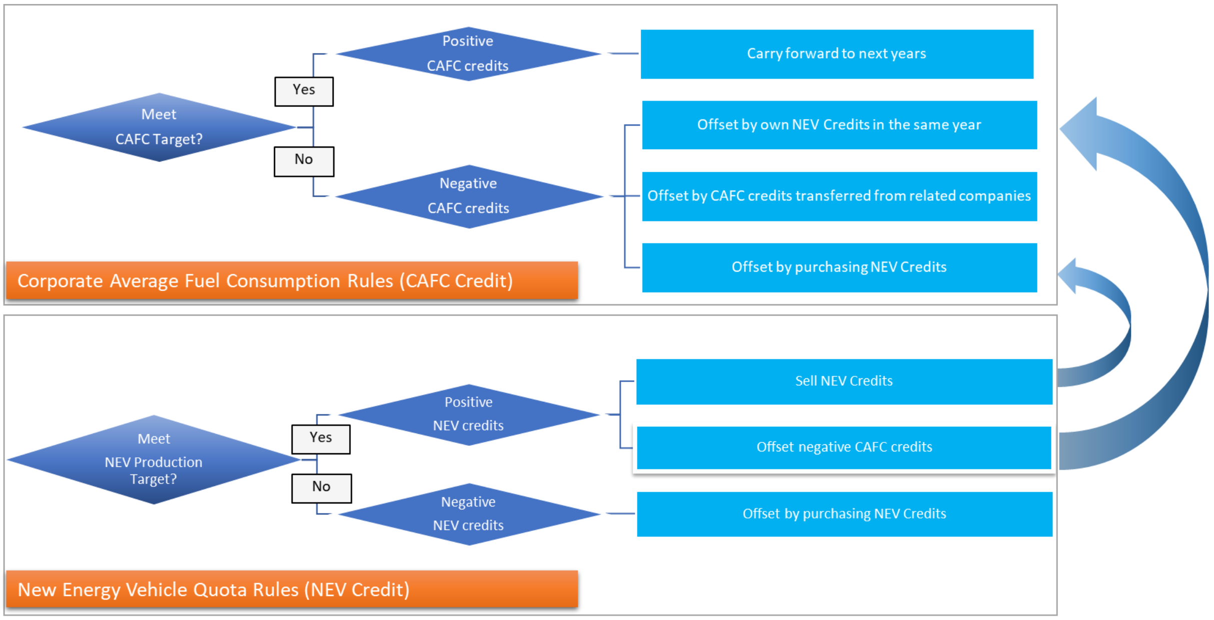

Supplementary Figure 1. Major rules in the Dual Credit policy

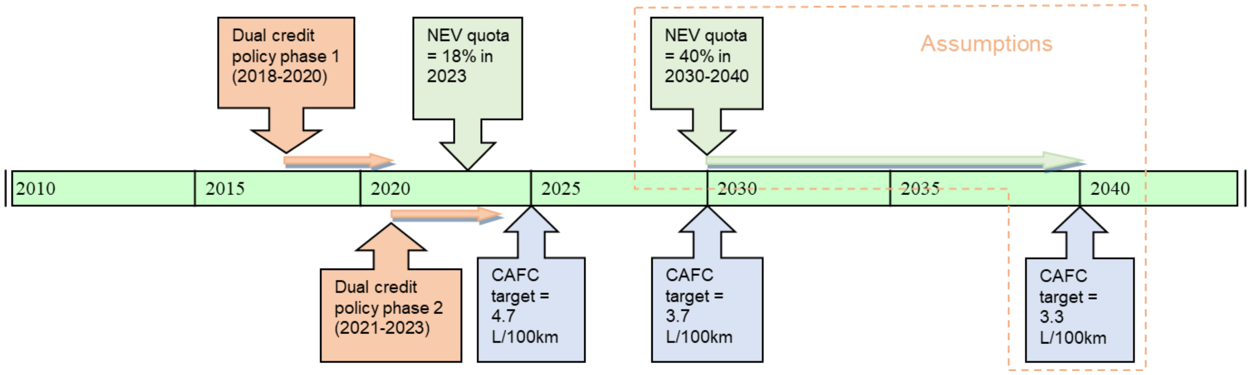

Supplementary Figure 2. Timeline of the existing Dual Credit policy and the future assumptions used in the NEOCC model

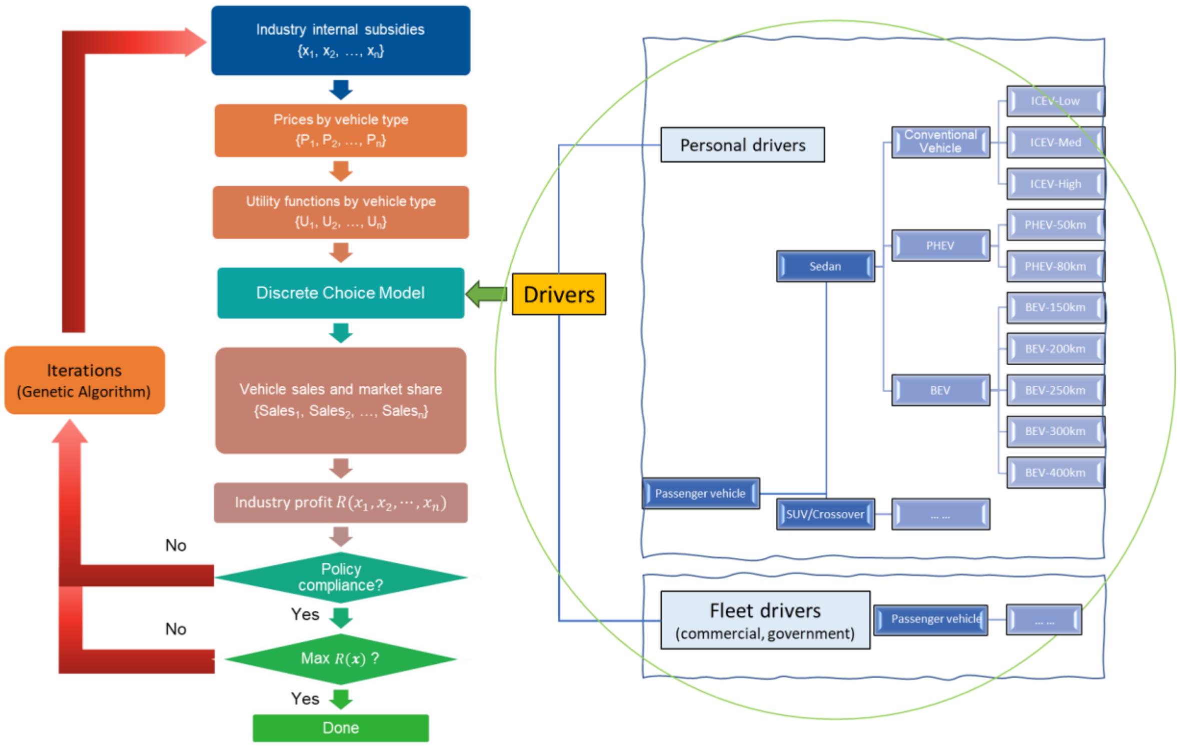

Supplementary Figure 3. NEOCC model algorithm logic flow.

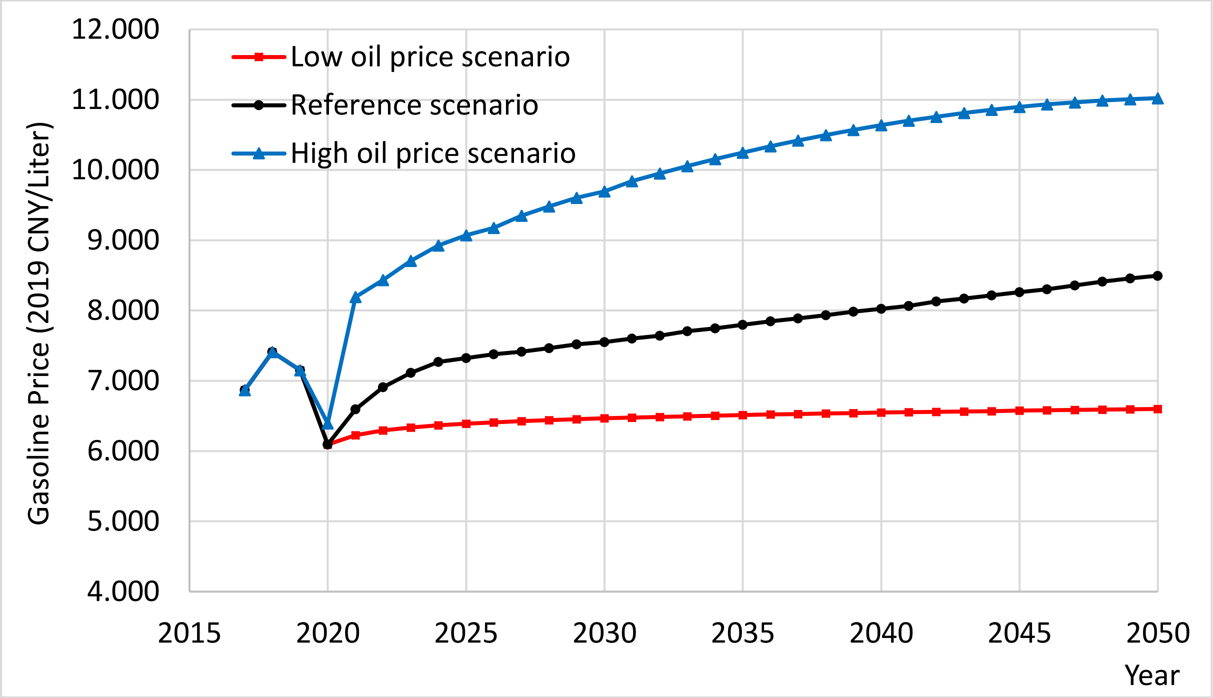

Supplementary Figure 4. Gasoline price projections for China under three international oil price scenarios

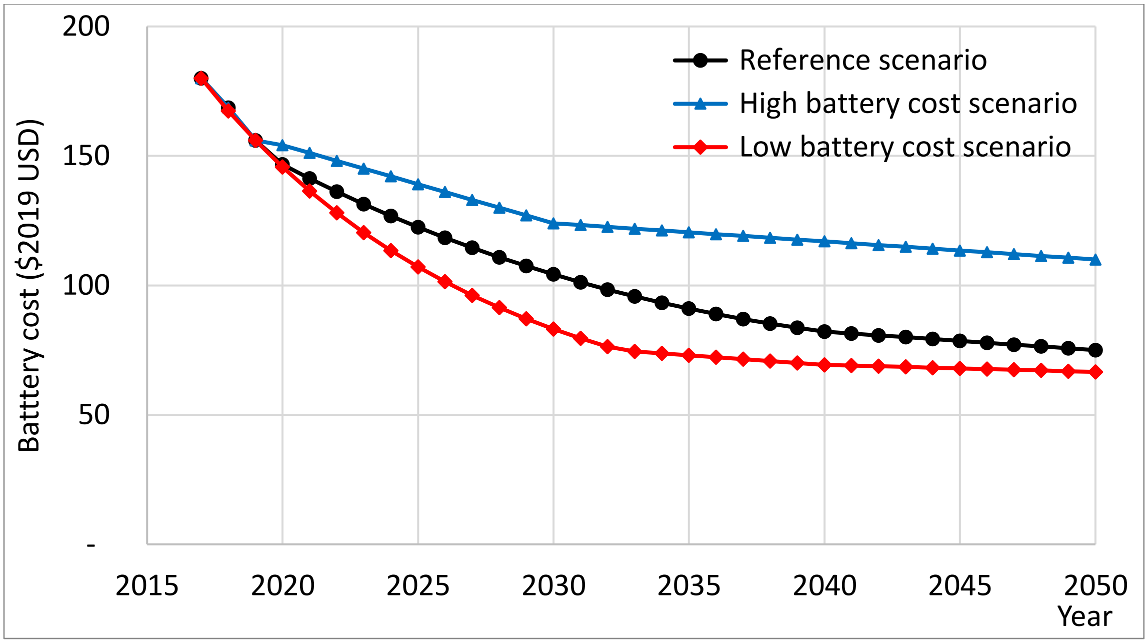

Supplementary Figure 5. Battery pack cost projections for three scenarios

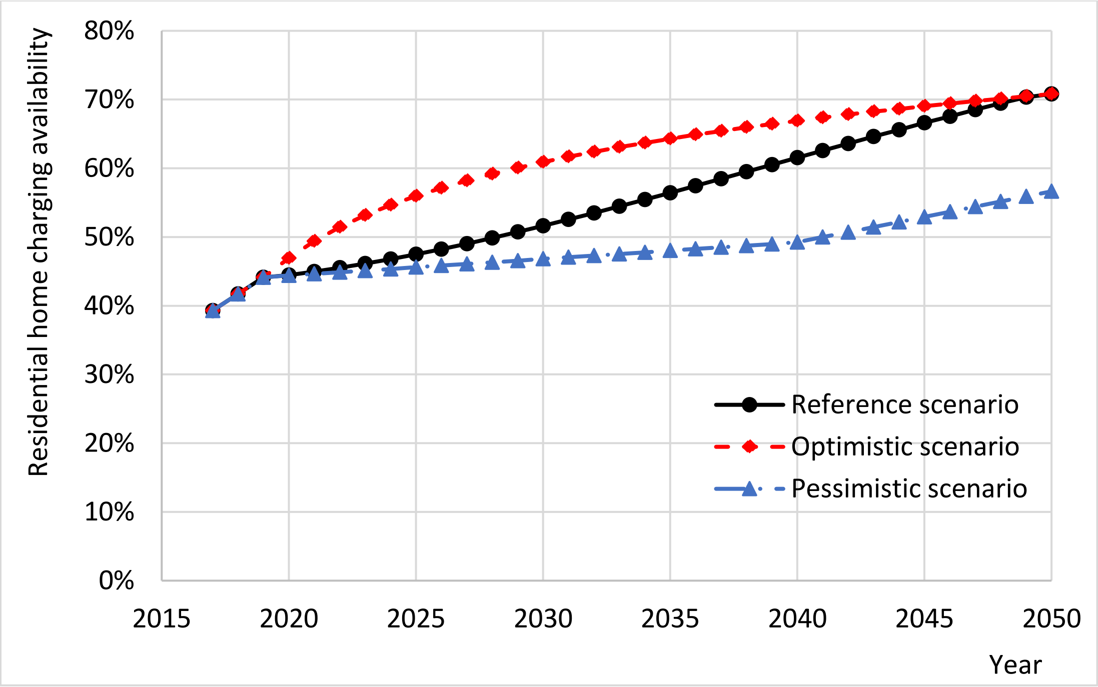

Supplementary Figure 6. Home charging availability projections for three scenarios

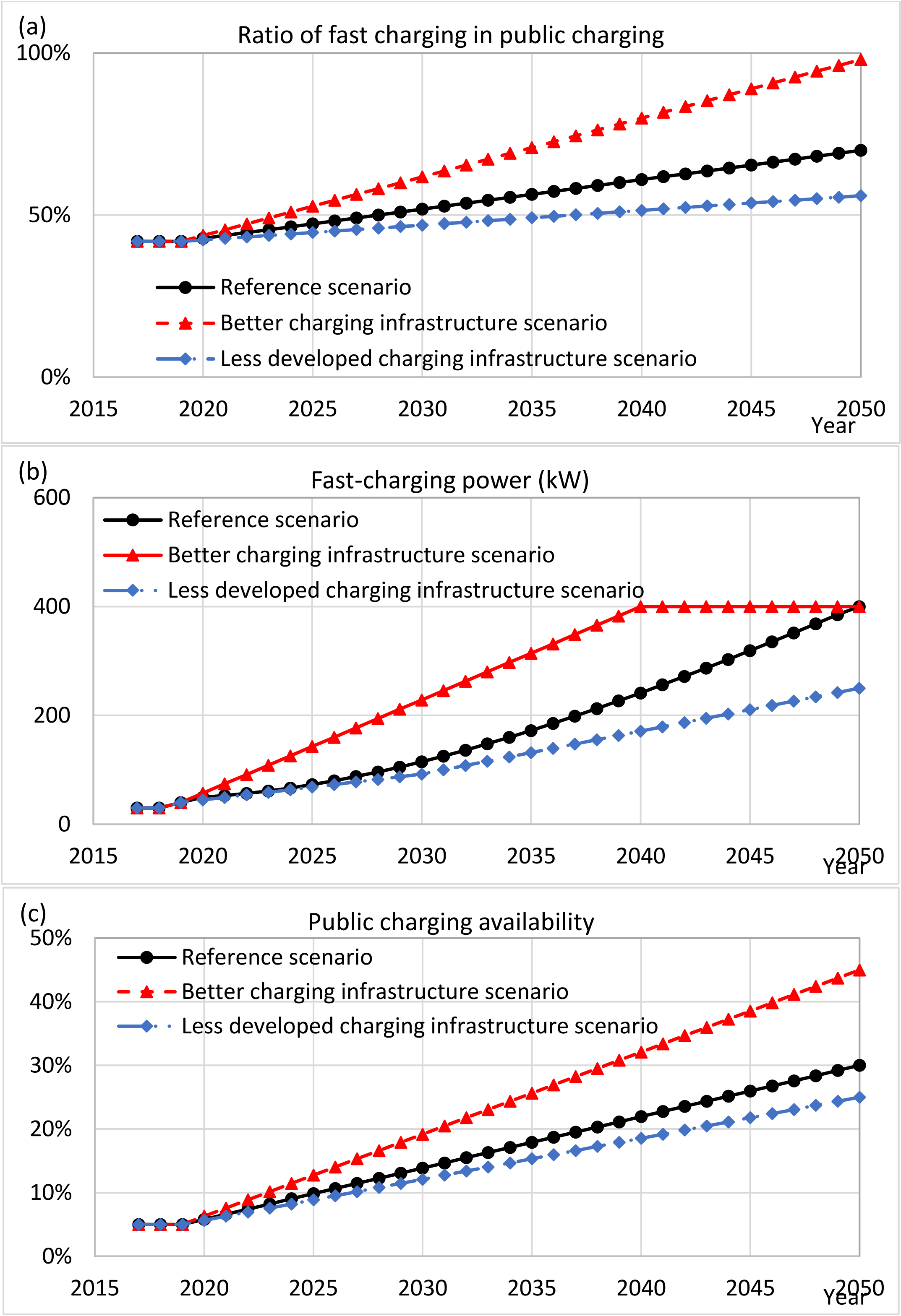

Supplementary Figure 7. Projection of public charging infrastructure parameters. (a) Ratio of fast charging in public charging; (b) Fast-charging power (kW); (c) Public charging availability.

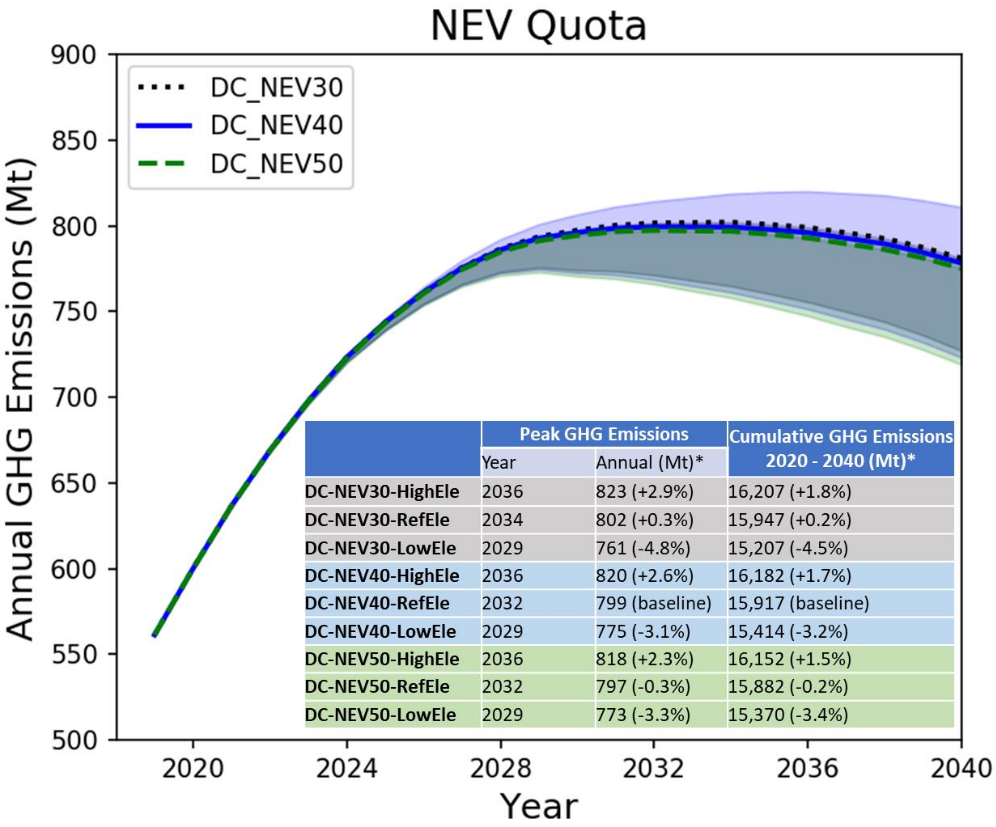

	Peak GHG Emissions		Cumulative GHG Emissions 2020 - 2040 (Mt)*
	Year	Annual (Mt)*	
**DC-NEV30-HighEle**	2036	823 (+2.9%)	16,207 (+1.8%)
**DC-NEV30-RefEle**	2034	802 (+0.3%)	15,947 (+0.2%)
**DC-NEV30-LowEle**	2029	761 (-4.8%)	15,207 (-4.5%)
**DC-NEV40-HighEle**	2036	820 (+2.6%)	16,182 (+1.7%)
**DC-NEV40-RefEle**	2032	799 (baseline)	15,917 (baseline)
**DC-NEV40-LowEle**	2029	775 (-3.1%)	15,414 (-3.2%)
**DC-NEV50-HighEle**	2036	818 (+2.3%)	16,152 (+1.5%)
**DC-NEV50-RefEle**	2032	797 (-0.3%)	15,882 (-0.2%)
**DC-NEV50-LowEle**	2029	773 (-3.3%)	15,370 (-3.4%)

Supplementary Figure 8. Uncertainties of GHG emissions due to the NEV quota. The shaded area represents the uncertainties due to the GHG intensity of the Chinese electric grid.

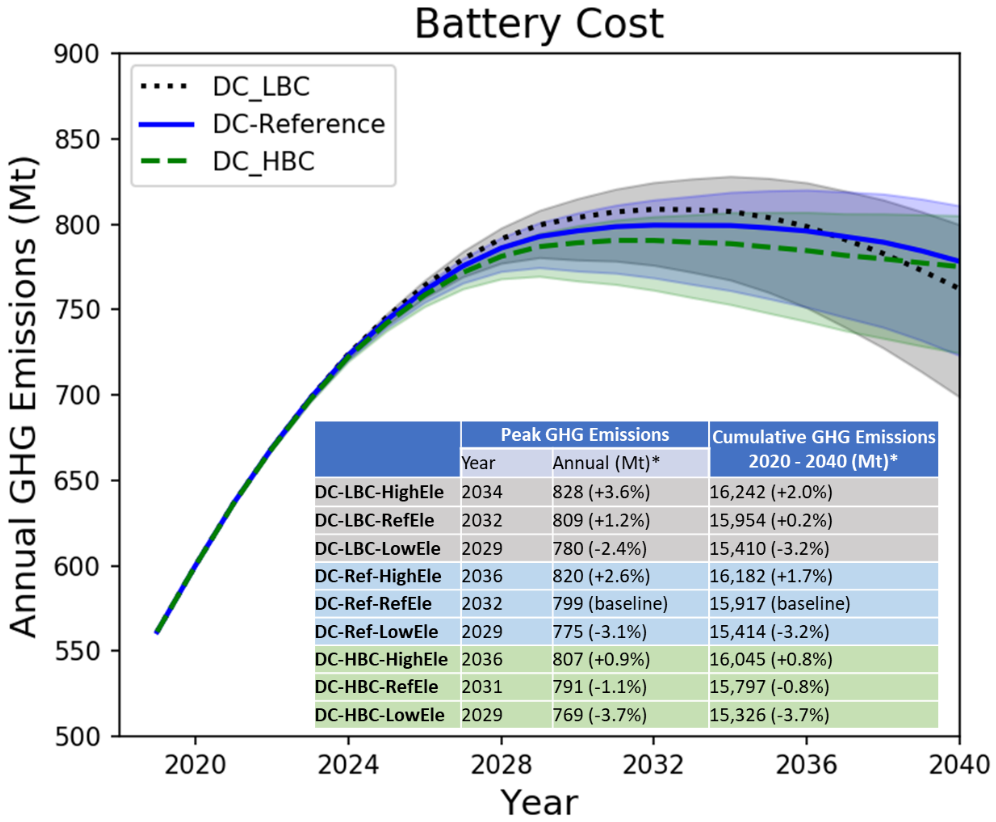

	Peak GHG Emissions		Cumulative GHG Emissions 2020 - 2040 (Mt)*
	Year	Annual (Mt)*	
**DC-LBC-HighEle**	2034	828 (+3.6%)	16,242 (+2.0%)
**DC-LBC-RefEle**	2032	809 (+1.2%)	15,954 (+0.2%)
**DC-LBC-LowEle**	2029	780 (-2.4%)	15,410 (-3.2%)
**DC-Ref-HighEle**	2036	820 (+2.6%)	16,182 (+1.7%)
**DC-Ref-RefEle**	2032	799 (baseline)	15,917 (baseline)
**DC-Ref-LowEle**	2029	775 (-3.1%)	15,414 (-3.2%)
**DC-HBC-HighEle**	2036	807 (+0.9%)	16,045 (+0.8%)
**DC-HBC-RefEle**	2031	791 (-1.1%)	15,797 (-0.8%)
**DC-HBC-LowEle**	2029	769 (-3.7%)	15,326 (-3.7%)

Supplementary Figure 9. Uncertainties of GHG emissions due to battery cost. The shaded area represents the uncertainties due to the GHG intensity of the Chinese electric grid.

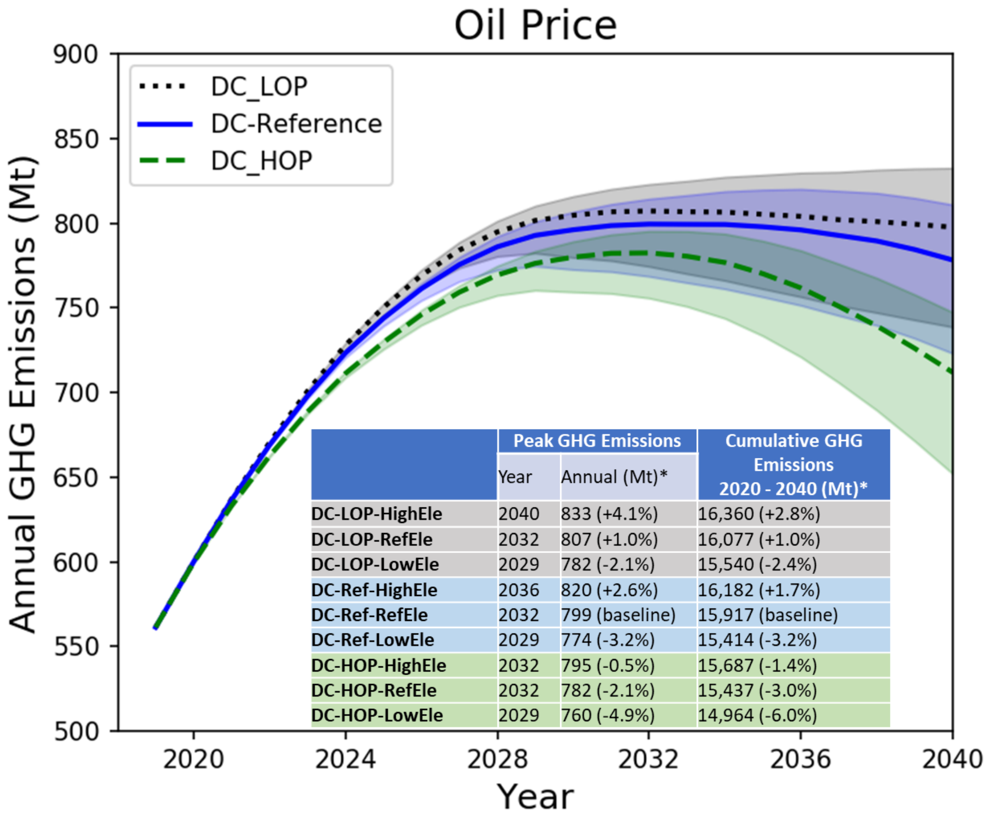

	Peak GHG Emissions		Cumulative GHG Emissions 2020 - 2040 (Mt)*
	Year	Annual (Mt)*	
**DC-LOP-HighEle**	2040	833 (+4.1%)	16,360 (+2.8%)
**DC-LOP-RefEle**	2032	807 (+1.0%)	16,077 (+1.0%)
**DC-LOP-LowEle**	2029	782 (-2.1%)	15,540 (-2.4%)
**DC-Ref-HighEle**	2036	820 (+2.6%)	16,182 (+1.7%)
**DC-Ref-RefEle**	2032	799 (baseline)	15,917 (baseline)
**DC-Ref-LowEle**	2029	774 (-3.2%)	15,414 (-3.2%)
**DC-HOP-HighEle**	2032	795 (-0.5%)	15,687 (-1.4%)
**DC-HOP-RefEle**	2032	782 (-2.1%)	15,437 (-3.0%)
**DC-HOP-LowEle**	2029	760 (-4.9%)	14,964 (-6.0%)

Supplementary Figure 10. Uncertainties of GHG emissions due to oil price. The shaded area represents the uncertainties due to the GHG intensity of the Chinese electric grid.

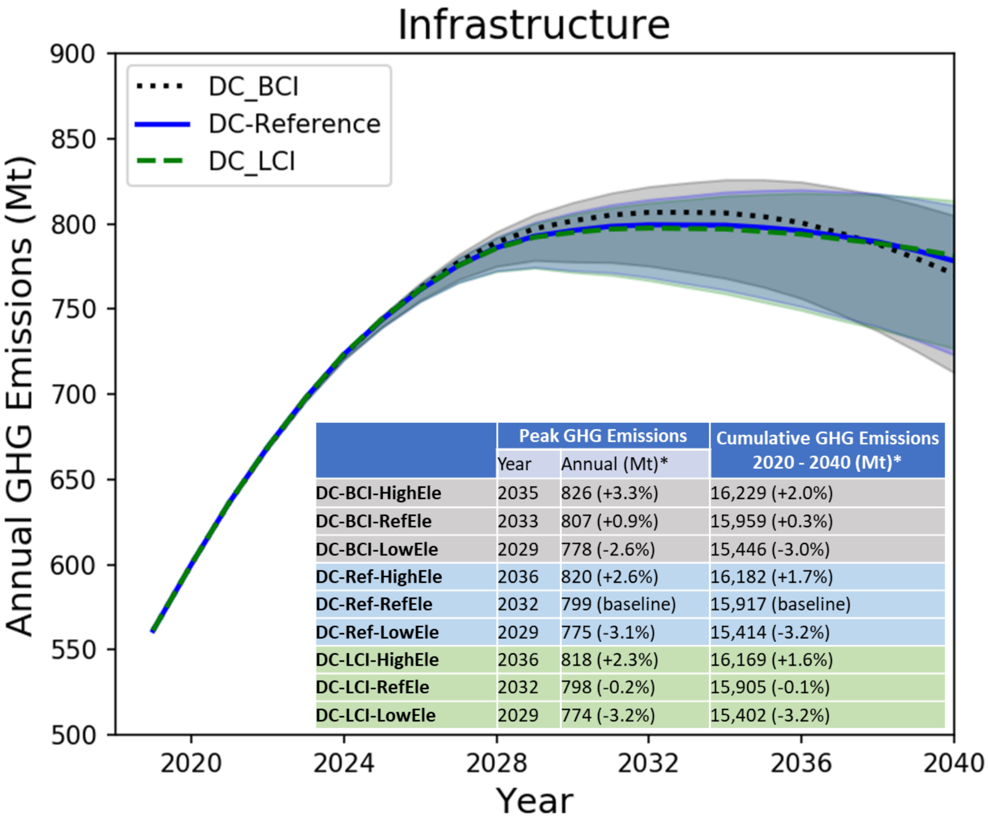

	Peak GHG Emissions		Cumulative GHG Emissions 2020 - 2040 (Mt)*
	Year	Annual (Mt)*	
**DC-BCI-HighEle**	2035	826 (+3.3%)	16,229 (+2.0%)
**DC-BCI-RefEle**	2033	807 (+0.9%)	15,959 (+0.3%)
**DC-BCI-LowEle**	2029	778 (-2.6%)	15,446 (-3.0%)
**DC-Ref-HighEle**	2036	820 (+2.6%)	16,182 (+1.7%)
**DC-Ref-RefEle**	2032	799 (baseline)	15,917 (baseline)
**DC-Ref-LowEle**	2029	775 (-3.1%)	15,414 (-3.2%)
**DC-LCI-HighEle**	2036	818 (+2.3%)	16,169 (+1.6%)
**DC-LCI-RefEle**	2032	798 (-0.2%)	15,905 (-0.1%)
**DC-LCI-LowEle**	2029	774 (-3.2%)	15,402 (-3.2%)

Supplementary Figure 11. Uncertainties of GHG emissions due to charging infrastructure. The shaded area represents the uncertainties due to the GHG intensity of the Chinese electric grid.

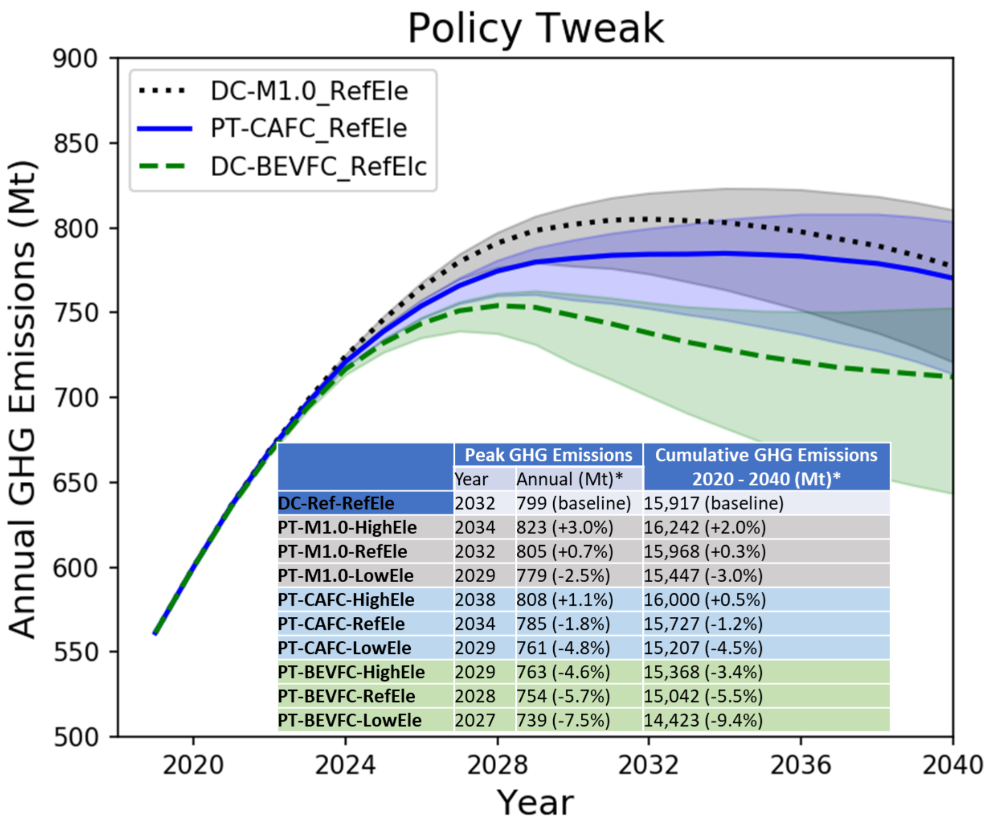

	Peak GHG Emissions		Cumulative GHG Emissions 2020 - 2040 (Mt)*
	Year	Annual (Mt)*	
**DC-Ref-RefEle**	2032	799 (baseline)	15,917 (baseline)
**PT-M1.0-HighEle**	2034	823 (+3.0%)	16,242 (+2.0%)
**PT-M1.0-RefEle**	2032	805 (+0.7%)	15,968 (+0.3%)
**PT-M1.0-LowEle**	2029	779 (-2.5%)	15,447 (-3.0%)
**PT-CAFC-HighEle**	2038	808 (+1.1%)	16,000 (+0.5%)
**PT-CAFC-RefEle**	2034	785 (-1.8%)	15,727 (-1.2%)
**PT-CAFC-LowEle**	2029	761 (-4.8%)	15,207 (-4.5%)
**PT-BEVFC-HighEle**	2029	763 (-4.6%)	15,368 (-3.4%)
**PT-BEVFC-RefEle**	2028	754 (-5.7%)	15,042 (-5.5%)
**PT-BEVFC-LowEle**	2027	739 (-7.5%)	14,423 (-9.4%)

Supplementary Figure 12. Uncertainties of GHG emissions due to the GHG intensity of the Chinese electric grid for policy tweak scenarios. The shaded area represents the uncertainties due to the GHG intensity of the Chinese electric grid.

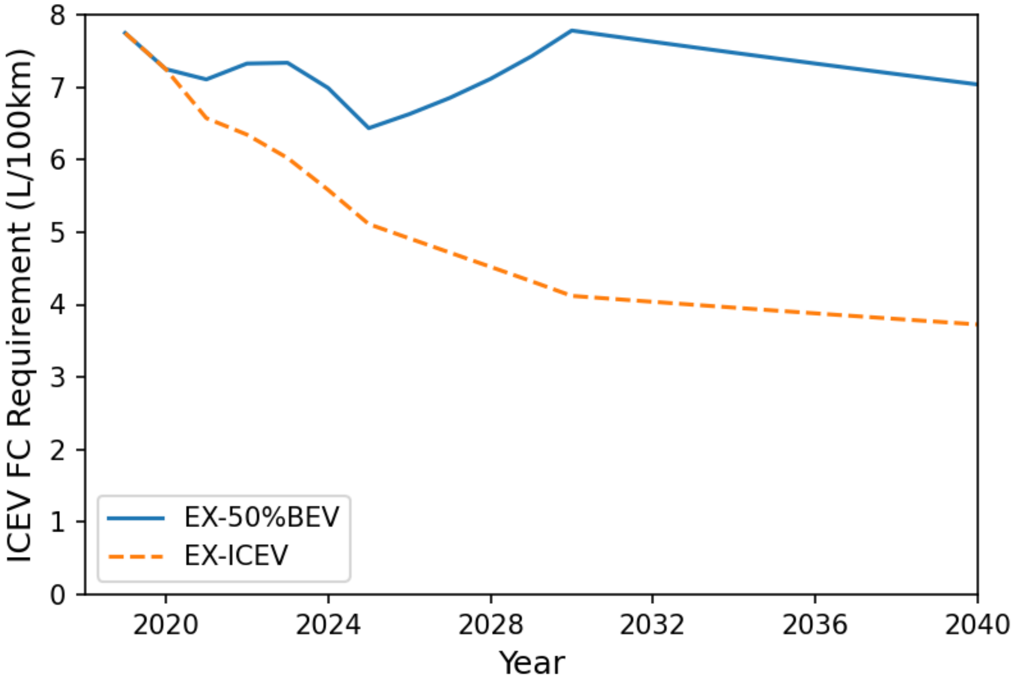

Supplementary Figure 13. Comparison of ICEV FC to meet the CAFC standards under EX-ICEV and EX-50%BEV scenarios

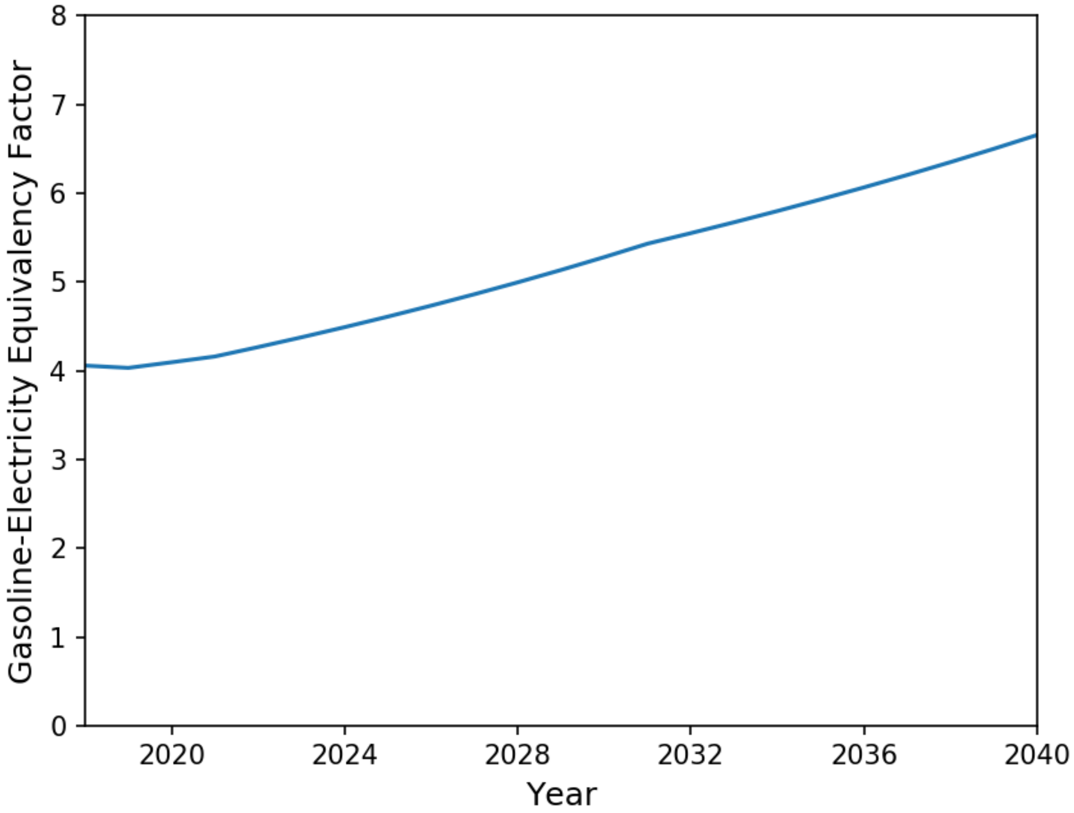

Supplementary Figure 14. Gasoline-Electricity Equivalency Factor

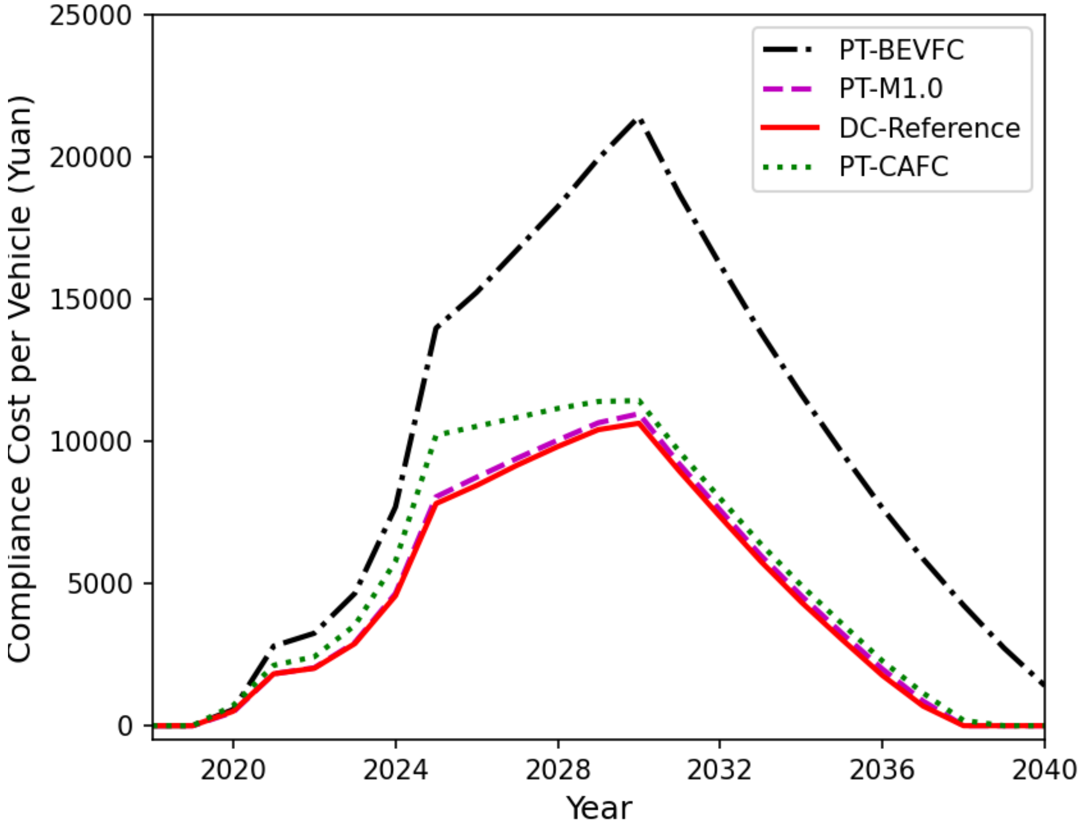

Supplementary Figure 15. The cost of regulation compliance under various policy tweak scenarios

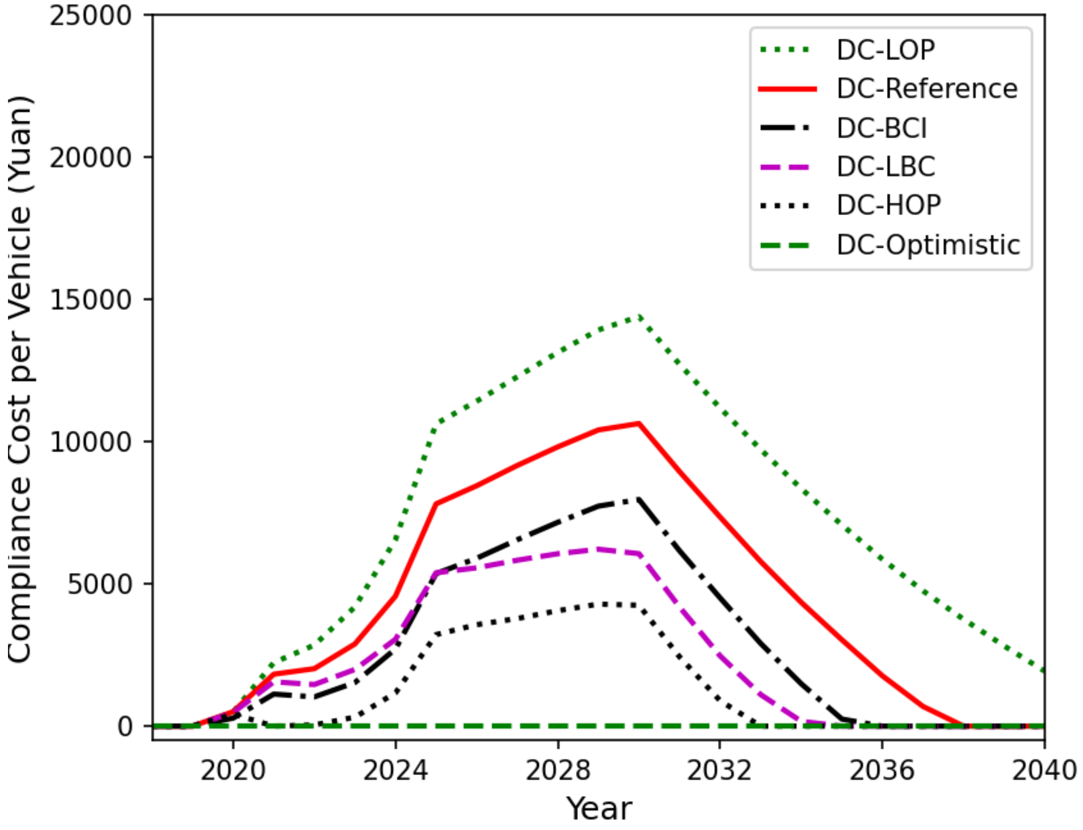

Supplementary Figure 16. The cost of regulation compliance under various externality scenarios

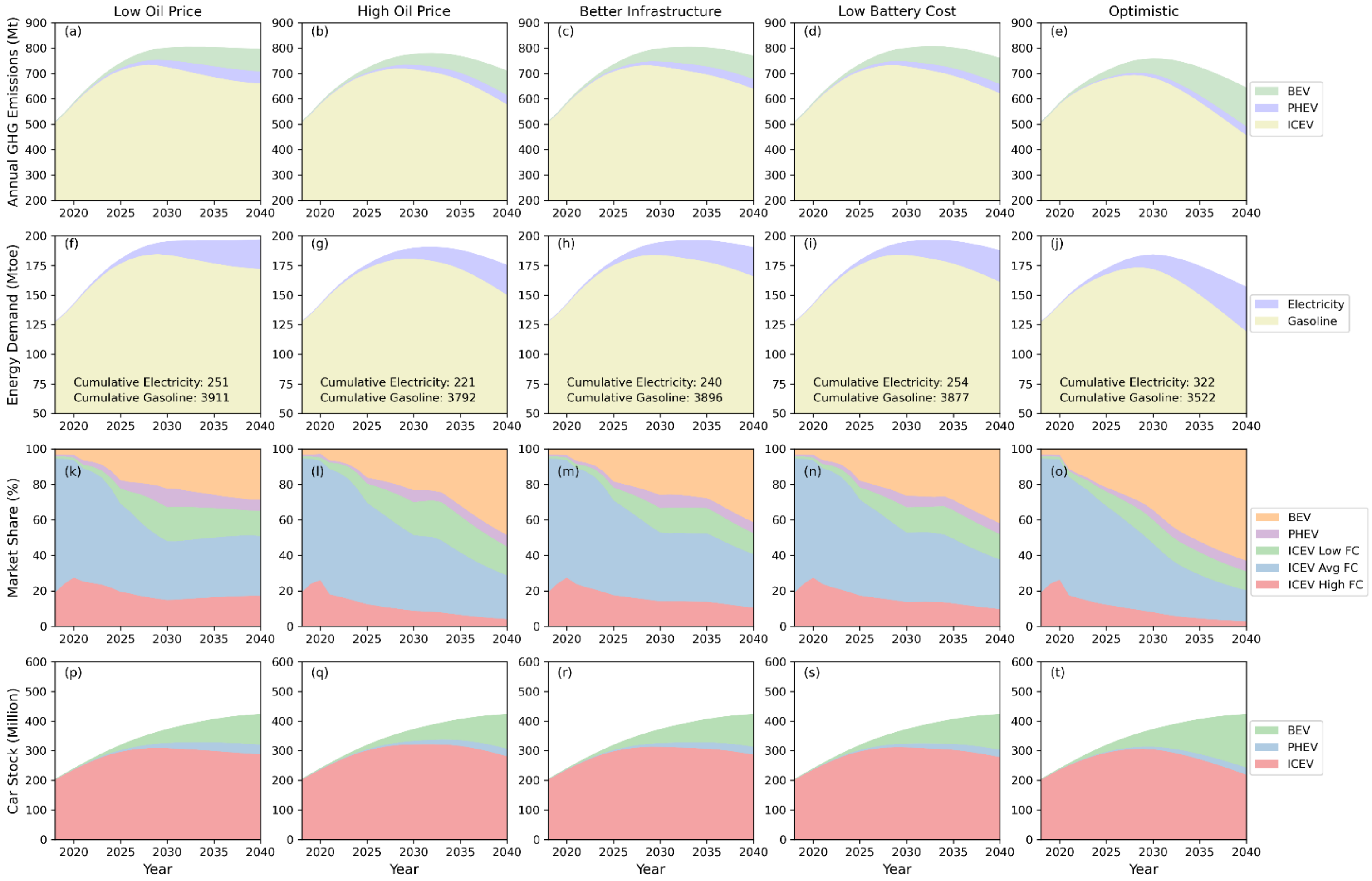

Supplementary Figure 17. Life-cycle GHG emissions breakdown to vehicle type, TTW energy demand, new sales market share and car stock under various externality scenarios. Annual GHG emissions: (a) DC-LOP; (b) DC-HOP; (c) DC-BCI; (d) DC-LBC; (e) DC-Optimistic. Energy demand: (f) DC-LOP; (g) DC-HOP; (h) DC-BCI; (i) DC-LBC; (j) DC-Optimistic. New sales market share: (k) DC-LOP; (l) DC-HOP; (m) DC-BCI; (n) DC-LBC; (o) DC-Optimistic. Total car stock: (p) DC-LOP; (q) DC-HOP; (r) DC-BCI; (s) DC-LBC; (t) DC-Optimistic.

Supplementary Tables

Supplementary Table 1. Summary of NEV credit calculation rules in the Dual Credit policy for 2018–2020

Technology	Electric range R (km)	Weight m (kg)	Fuel Consumption FC (L/100km)	Electric Consumption Y (kWh/100km)	Battery Rated Power P (kW)	Scores	Multiplier	Are Credits Tradable? (Y/N)
PHEV/EREV	50≤R<80		FC < 0.7× (GBT 19578-2014 Test B)			=2	×1.0	Y
			FC ≥ 0.7× (GBT 19578-2014 Test B)				×0.5	N
	R≥80	m≤1000		Y ≤ 0.014×m+0.5			×1.0	Y
				Y > 0.014×m+0.5			×0.5	N
		1000<m≤1600		Y ≤ 0.012×m+2.5			×1.0	Y
				Y > 0.012×m+2.5			×0.5	N
		m>1600		Y ≤ 0.005×m+13.7			×1.0	Y
				Y > 0.005×m+13.7			×0.5	N
BEV	R≥100	m≤1000		Y>0.014×m+0.5		= 0.012×R +0.8, and ≤ 5	×0.5	N
				0.0098×m+0.35 < Y ≤ 0.014×m+0.5			×1.0	Y
				Y ≤ 0.0098×m+0.35			×1.2	Y
		1000<m≤1600		Y>0.012×m+2.5			×0.5	N
				0.0084×m+1.75 < Y ≤ 0.012×m+2.5			×1.0	Y
				Y ≤ 0.0084×m+1.75			×1.2	Y
		m>1600		Y>0.005×m+13.7			×0.5	N
				0.0035×m+9.59 < Y ≤ 0.005×m+13.7			×1.0	Y
				Y ≤ 0.0035×m+9.59			×1.2	Y
FCEV	R≥300				P≥30% × (Motor power), and ≥10 kW	= 0.16×P	×1.0	Y
							×0.5	N

Supplementary Table 2. Summary of NEV credit calculation rules in the Dual Credit policy for 2021–2023 (according to the version released for public comment)

Technology	Electric range R (km)	Weight m (kg)	Fuel Consumption FC (L/100km)	Electric Consumption Y (kWh/100km)	Battery Rated Power P (kW)	Scores	Multiplier	Are Credits Tradable? (Y/N)
PHEV/EREV		m≤1000	FC < 0.7× (GBT 19578-2014 Test B)	Y ≥ 1.35×(0.0112×m+0.4)		=1.6	×0.5	N
				Y < 1.35×(0.0112×m+0.4)			×1.0	Y
		1000<m≤1600		Y ≥ 1.35×(0.0078×m+3.8)			×0.5	N
				Y < 1.35×(0.0078×m+3.8)			×1.0	Y
		m>1600		Y ≥ 1.35×(0.0044×m+9.24)			×0.5	N
				Y < 1.35×(0.0044×m+9.24)			×1.0	Y
			FC ≥ 0.7× (GBT 19578-2014 Test B)				×0.5	N
BEV	100≤R<150	m≤1000		Y > 0.0112×m+0.4		=1.0	×0.5	N
				Y ≤ 0.0112×m+0.4			×1.0	Y
		1000<m≤1600		Y > 0.0078×m+3.8			×0.5	N
				Y ≤ 0.0078×m+3.8			×1.0	Y
		m>1600		Y > 0.0044×m+9.24			×0.5	N
				Y ≤ 0.0044×m+9.24			×1.0	Y
	R≥150	m≤1000		Y > 0.0112×m+0.4		= 0.006×R +0.4, and ≤ 3.4	×0.5	N
				Y ≤ 0.0112×m+0.4			×1.0	Y
		1000<m≤1600		Y > 0.0078×m+3.8			×0.5	N
				Y ≤ 0.0078×m+3.8			×1.0	Y
		m>1600		Y > 0.0044×m+9.24			×0.5	N
				Y ≤ 0.0044×m+9.24			×1.0	Y
FCEV	R≥300				P≥30% × (Motor power), and ≥10 kW	= 0.08×P, and ≤ 6	×1.0	Y
							×0.5	N

Supplementary Table 3. General information of 2016–2019 passenger vehicle market in China.

Class	Type	Sales	VCW (kg)	Electric Range (km)	FC-Charge Sustaining (L/100 km)	EC-Charge Depleting (kWh/100 km)	Price ($2019)
**Market in 2016:**							
Sedan	ICEV-High	3,848,546	1,303	-	8.56	-	144,535
	ICEV-Med	8,202,637	1,303	-	6.36	-	153,474
	ICEV-Low	169,641	1,303	-	5.00	-	169,339
	PHEV-50	44,097	1,749	63	5.35	16.51	344,531
	PHEV-80	2	1,904	85	6.60	20.50	347,140
	BEV-150	124,093	1,084	150	-	13.97	202,347
	BEV-200	40,882	1,494	200	-	16.74	238,317
	BEV-250	1,819	1,498	250	-	16.45	263,598
	BEV-300	40,882	1,810	300	-	16.94	299,544
	BEV-400	-	2,407*	400*	-	20.50*	401,061
SUV/ Cross-over	ICEV-High	1,845,306	1,536	-	10.31	-	176,719
	ICEV-Med	7,002,408	1,536	-	7.92	-	181,486
	ICEV-Low	16,236	1,536	-	6.06	-	193,587
	PHEV	26,497	1,882	85	8.75	24.57	366,039
	BEV-250	8,783	1,298	200	-	18.77	335,091
	BEV-350	279	1,486	300	-	20.63	396,472
**Market in 2017:**							
Sedan	ICEV-High	4,232,349	1,321	-	8.07	-	153,074
	ICEV-Med	6,904,604	1,321	-	6.07	-	163,063
	ICEV-Low	159,693	1,321	-	4.61	-	190,748
	PHEV-50	23,592	1,627	58	5.31	13.41	303,035
	PHEV-80	16,596	1,756	84	5.32	17.78	326,389
	BEV-150	218,153	987	158	-	12.73	139,484
	BEV-200	134,299	1,466	201	-	15.26	173,881
	BEV-250	20,242	1,480	254	-	14.93	197,429
	BEV-300	23,838	1,788	315	-	15.38	231,629
	BEV-400	7,010	2,407	400*	-	15.00*	269,459
SUV/ Cross-over	ICEV-High	2,717,363	1,525	-	9.98	-	155,243
	ICEV-Med	9,314,814	1,525	-	7.57	-	161,129
	ICEV-Low	22,186	1,788	-	6.06	-	171,584
	PHEV	58,070	2,017	74	5.94	17.00	315,081
	BEV-250	28,624	1,732	250	-	16.28	356,585
	BEV-350	19,883	1,982	350	-	17.89	423,288
**Market in 2018:**							
Sedan	ICEV-High	1,829,787	1,352	-	9.38	-	162,359

	ICEV-Med	8,383,391	1,352	-	6.03	-	173,887
	ICEV-Low	199,930	1,352	-	4.83	-	191,562
	PHEV-50	66,424	1,664	56	1.82	14.11	306,417
	PHEV-80	40,612	1,750	84	1.31	17.20	326,018
	BEV-150	61,869	976	158	-	12.71	151,646
	BEV-200	115,382	1,037	206	-	12.70	169,940
	BEV-250	107,927	1,098	256	-	12.73	189,790
	BEV-300	131,210	1,303	310	-	13.16	214,873
	BEV-400	106,477	1,736	404	-	14.42	268,065
SUV/ Cross-over	ICEV-High	2,385,512	1,545	-	9.61	-	179,078
	ICEV-Med	8,172,934	1,545	-	7.48	-	184,431
	ICEV-Low	56,726	1,545	-	5.85	-	195,349
	PHEV	110,774	2,047	71	1.64	17.80	336,920
	BEV-250	28,580	1,525	250	-	14.79	226,603
	BEV-350	159,942	1,745	350	-	16.26	284,191
**Market in 2019:**							
Sedan	ICEV-High	2,810,372	1,357	-	8.19	-	155,372
	ICEV-Med	6,551,233	1,357	-	5.82	-	165,606
	ICEV-Low	216,995	1,357	-	4.66	-	185,139
	PHEV-50	96,919	1,625	60	1.49	16.00	235,995
	PHEV-80	10,142	1,764	83	1.04	17.20	249,488
	BEV-150	902	966	153	-	10.00	130,007
	BEV-200	11,561	1,096	203	-	10.46	146,964
	BEV-250	53,900	1,319	254	-	10.73	163,922
	BEV-300	128,516	1,400	307	-	11.67	186,531
	BEV-400	290,410	1,603	414	-	13.70	245,882
SUV/ Cross-over	ICEV-High	2,135,187	1,621	-	9.64	-	168,558
	ICEV-Med	7,890,286	1,621	-	7.22	-	174,775
	ICEV-Low	78,081	1,621	-	5.65	-	185,212
	PHEV	85,657	2,016	71	1.65	16.46	260,749
	BEV-250	2,391	1,620	263	-	15.00	243,069
	BEV-350	182,073	1,854	365	-	16.49	299,593

*** The weights are adjusted based on the most popular model of this vehicle type.**

Supplementary Table 4. Updates and adjustments in the Base Case of China Vehicle Fleet Model.

Year	Sales (thousands of vehicle)			Stocks (thousands of vehicle)			Fleet Average VKT per vehicle (1000 km/year)
	private LDPV	taxi	business LDPV	private LDPV	taxi	LDPV	
2018	23000	316	2095	189000	1393	11939	10.79
2019	21271	320	1971	208408	1467	13117	10.59
2020	22405	324	1855	225298	1492	14110	10.56
2021	23459	328	1748	241927	1576	14938	10.54
2022	24436	332	1651	258020	1619	15632	10.53
2023	25340	336	1565	273343	1640	16231	10.55
2024	26164	341	1488	287727	1661	16778	10.59
2025	26903	346	1422	301060	1683	17306	10.67
2026	27551	350	1366	313281	1705	17827	10.79
2027	28144	355	1322	324414	1728	18330	10.94
2028	28688	360	1289	334552	1752	18779	11.10
2029	29135	365	1267	343808	1776	19130	11.27
2030	29436	369	1252	352295	1800	19348	11.44
2031	29478	373	1265	360005	1823	19440	11.62
2032	29589	377	1308	367202	1845	19442	11.78
2033	29742	380	1352	374017	1865	19381	11.94
2034	29906	383	1368	380460	1883	19265	12.10
2035	29850	386	1355	386259	1900	19101	12.25
2036	29762	389	1333	391329	1916	18912	12.40
2037	29692	392	1304	395659	1931	18713	12.54
2038	29690	394	1270	399316	1944	18511	12.70
2039	29771	396	1223	402412	1957	18299	12.84
2040	29599	398	1162	404741	1969	18063	12.99
2041	29787	400	1101	406725	1980	17798	13.14
2042	29875	402	1054	408335	1990	17510	13.27
2043	29879	404	1017	409562	2001	17203	13.39
2044	29846	407	980	410444	2011	16869	13.51
2045	29763	409	945	411000	2022	16503	13.62
2046	29619	412	916	411229	2034	16107	13.72
2047	29410	414	893	411115	2046	15689	13.80
2048	29144	417	874	410645	2059	15255	13.88
2049	28822	420	859	409796	2071	14817	13.95
2050	28446	422	848	408542	2084	14384	14.02

Supplementary Notes

Supplementary Note 1. Corporate Average Fuel Consumption (CAFC) policy, Dual Credit policy, and future assumptions

The CAFC target and the actual CAFC for each automaker are calculated using the production-weighted expected specific fuel consumption (FC) based on the vehicle curb weight (VCW) and the actual production-weighted specific fuel economy, respectively. Stage 4 of GB 27999-2014 (the Chinese standard) defines the CAFC standard for model years (MYs) 2016–2020. The Ministry of Industry and Information Technology (MIIT) recently released an opinion-soliciting draft of "Fuel Consumption Evaluation Methods and Targets for Passenger Cars," [1] which will eventually define the Stage 5 CAFC target. Notably, Stage 4 uses the New European Driving Cycle (NEDC) as the standard driving cycle in evaluating vehicle FC, while Stage 5 uses the Worldwide Harmonized Light Vehicle Test Procedure (WLTP) cycle. The regression analysis, based on the test results of the selected representative vehicles in China, has shown that, on average, vehicles driven on the WLTP cycle emit about 10.5% more CO_2 emissions than when driven on the NEDC cycle [2]. To be consistent, the CAFC targets before 2021 are adjusted to the WLTP cycle by multiplying the NEDC-based target by a factor of 1.105. Adjusted by vehicle sales (this study assumes the production and sales volumes are equivalent) and VCW, the WLTP-based CAFC target in 2025 will be 4.7 L/100 km. The Stage 4 and Stage 5 CAFC standards set the FC of BEVs to be zero. Meanwhile, the production multipliers of 2.0, 1.8, 1.6, and 1.3 are applied to BEV MYs from 2021 to 2024, respectively.

The Dual Credit policy consists of two components: CAFC credit rules and NEV credit rules. The CAFC credit rules set production-weighted average FC targets for vehicle manufacturers, and the NEV credit rules mandate that manufacturers produce enough NEVs to meet the NEV credit quota [3]. Meanwhile, the Dual Credit policy allows manufacturers to use surplus NEV credits to compensate for CAFC deficits if the CAFC target is not achieved. In 2019 and 2020, the NEV quota is set to 10% and 12%, respectively. The number of NEV credits granted to each NEV varies depending on vehicle powertrain type, VCW, electric driving range, and vehicle energy efficiency, as shown in Supplementary Tables

Supplementary Table 1. A maximum of 5 credits can be granted to an NEV. In September 2019, MIIT released the second phase of the Dual Credit policy for MY 2021–2023, [1] elevating the NEV credit requirements. First, the NEV quota increases to 14%, 16%, and 18% in 2021–2023, respectively. Second, the number of credits granted to each NEV is almost halved, as shown in Supplementary Table 2. Third, in order to stimulate the development of fuel-efficient internal combustion engine vehicles (ICEVs), a multiplier of 0.5 is applied in calculating the number of fuel-efficient ICEVs in the Dual Credit Policy for MY 2021-2023; it means fewer NEV credits are needed to meet the target. The Dual Credit Policy defines the fuel-efficient ICEVs as vehicles with FC lower than the CAFC target.

To meet the CAFC credit rules, the actual CAFC of an auto firm should equal the target CAFC. The CAFC credits are calculated based on the production volume-weighted of the differences between the actual CAFC and target CAFC in vehicle models [3]. To meet the NEV credit requirements, the ratio of NEVs to ICEVs produced or imported by an auto firm should be larger than the required quota. The NEV credits are calculated based on the difference between achieved NEV scores and the NEV minimum ratios [3]. The detailed calculation methods for NEV scores for different powertrain types are given by Supplementary Tables

Supplementary Table 1 and Supplementary Table 2. The Dual Credit policy also allows for a 1:1 transfer of NEV credits to CAFC credits to compensate for any potential deficits in CAFC credits. The NEV credits can be traded among companies, and CAFC credits can be carried forward for future use. The relationship between CAFC credit rules and NEV credit rules are explained by Supplementary Figure 1. To avoid potential penalties by the government, the auto companies must meet both the CAFC and NEV targets.

Supplementary Figure 2 shows the timeline of the Dual Credit policy used in this study. The existing Dual Credit and CAFC standards are defined for FC targets until 2023 and 2025, respectively. Beyond the defined target dates, the following assumptions are made: First, the CAFC target decreases linearly from 4.7 to 3.7 L/100 km from 2025 to 2030, 4% per year on average over a 5-year period. This assumption is based on the vehicle fuel economy improvements proposed by the Chinese Society of Automotive Engineers (SAE-China) in *Technology Roadmap for Energy Saving and New Energy Vehicles* [4]. Since no official CAFC targets for years after 2030 have been released by policymakers, we assume a moderate CAFC reduction of 1% per year, reaching 3.3 L/100 km by 2040. Second, the NEV quota assumes a linear increase from 18% in 2023 to 40% in 2030. After evaluating a series of the government policies published in recent years and discussing the issue with experts and policymakers in China, we believe a 40% NEV quota is feasible for 2030. The rules in Dual Credit policy become more favorable to promote BEV efficiency instead of longer electric driving range. Therefore, in the model, the number of NEV credits granted to each NEV is assumed to decrease linearly to 1.0 by 2030. At last, all other parts of the policy remain the same as the last draft of the standards as defined in CAFC and Dual Credit policies.

Supplementary Note 2. Details of the NEOCC model

(a) NEOCC model algorithm logic flow

Supplementary Figure 3 shows the NEOCC model algorithm logic flow. The industry internal subsidies work as the decision variables in the NEOCC model, and the allocations of these subsidies will change the total utilities and purchase probabilities of the different vehicle types [3]. Drivers in the passenger vehicle market are classified into two segments: personal vehicle drivers and fleet drivers (including government vehicles and commercial vehicles).

(b) Model Calibration

The NEOCC model is calibrated based on the summarized Chinese passenger vehicle market information for 2016–2019. The market information is summarized in Supplementary Table 3. The vehicle parameters, such as VCW, electric range, FC-charge sustaining, EC-charge depleting, and price, are weighted by the vehicle models and their sales. The data analyses are supported by the China Automotive Technology and Research Center [6].

(c) Gasoline price projection

Three different oil price scenarios are studied: a reference scenario, a high oil price scenario, and a low oil price scenario. The statistical relationship between the retail gasoline prices in China and international crude oil prices is quantified by using the time-series error correction model [7]. As shown in Supplementary Figure 4, we project the retail gasoline prices in China from 2020 to 2050 based on three international crude oil price scenarios (reference scenario, high oil price scenario, and low oil price scenario) published in the U.S. Energy Information Administration's "Annual Energy Outlook 2020".

(d) Incremental cost of improving ICEV efficiency

The production cost of an ICEV increases when the vehicle is equipped with better fuel-saving technologies. In this study, the estimation of the incremental production cost of improving fuel economy is based on the Nissan Note (a compact car) and the Nissan Serena (a minivan)—both are currently sold in Japan [8,9]. The two vehicle models have a conventional ICEV version and an E-Power serial hybrid version, sold at a price difference of $4,475 for the Note [8] and $3,852 for the Serena [9]. Under the JP08 driving cycle, the differences in FC between the conventional ICEV and hybrid are 1.59 L/100 km (Note) and 2.0 L/100 km (Serena). When considering the FC difference in the JP08 cycle and in the WLTP cycle, the incremental cost of FC is adjusted to $2,320/(L/100 km). We assume the majority of Chinese OEMs will use a similar level of fuel-saving technology by 2030.

(e) Battery pack cost

Three scenarios are assumed for the battery pack cost: a reference scenario, a low battery cost scenario, and a high battery cost scenario. The projection of battery pack costs in some key years are based on the following literature review. Nykvist et al. (2018) estimated that the battery pack cost ranges from 210 to 230 USD/kWh in 2017 [10], and Kittrer et al. (2017) estimated the battery pack price to be $178/kWh in 2017 [11]. Correspondingly, this study assumes that the average battery pack cost in China is 180 USD/kWh in 2017 and 156 USD/kWh in 2019 [12]. By 2020, the industry experts believe battery pack cost can decrease to 124–175 USD/kWh [11,13]. Therefore, this study assumes the battery pack cost to be at a mid-point range of 147 USD/kWh. According to the "Technology Roadmap for Energy Saving and New Energy Vehicles" released by the Strategic Advisory Committee for the Chinese government and SAE-China, the report projects that battery pack cost could be at around 130 USD/kWh in 2025 and 116 USD/kWh in 2030 [4]. ICCT estimates that battery pack costs will decline to 120–135 USD/kWh by 2025 [14].

Furthermore, the electric vehicle battery goals by the U.S. Department of Energy indicate that the ideal battery pack cost is 80 USD/kWh. We assume this cost can be reached by 2040 and that it will decrease to 75 USD/kWh by 2050. In the most aggressive projection, Bloomberg New Energy Finance believes battery pack cost will fall to 100 USD/kWh by 2025 [15] and will continuously drop to 74 USD/kWh by 2030 [16]. However, MIT researchers feel less optimistic: they don't expect battery cost to reach 124 USD/kWh until 2030 [17]. Therefore, combining the assumption that China will continue to have a cheaper manufacturing cost than the U.S., this study assumes the following:

- Reference scenario: Battery pack cost will be near 120 USD/kWh in 2025, 104 USD/kWh in 2030, and 75 USD/kWh in 2050.
- Low battery cost scenario: Battery pack cost will be around 83 USD/kWh in 2030 and around 67 USD/kWh in 2050.
- High battery cost scenario: Battery pack cost will be around 124 USD/kWh in 2030 and around 110 USD/kWh in 2050.

For simplification, the battery pack costs in other years are linearly interpolated. The battery cost projections under the reference scenario, low battery cost scenario, and high battery cost scenario are shown in Supplementary Figure 5.

(f) Home charging availability

Supplementary Figure 6 shows the projected average availability of residential parking for NEV home charging in China from 2017 to 2050: reference scenario, optimistic scenario (better charging infrastructure), and pessimistic scenario (less developed charging infrastructure). Home charging is widely considered the most important charging option for NEV adoption, and adequate dedicated residential parking spaces are a prerequisite. However, unlike most new car buyers in the U.S., Chinese car owners predominantly live in high-rise apartments where parking spaces are limited. Even in megacities with both private and public parking spaces, the ratio of parking spaces to vehicles is only about 0.8 [18]. Recognizing the potential obstacles of residential parking space availability and quantifying the ownership impacts resulting from this availability is critical to understanding the actual NEV demand. We use estimates of current residential parking availability and future projections of home charging availability on the basis of government planning in the residential parking availability based on analyses by Ou et al. [18] By 2050, the residential availability of home parking may reach nearly 90% (i.e., 90% households can own at least one residential parking space, an ideal situation as the U.S. [18]). In addition, this study assumes that 80% of the residential parking availability can be converted to home charging. The following scenarios for charging infrastructure are used:

- Reference scenario: Home charging availability will be 56% by 2025 and 70.8% by 2050.
- Optimistic scenario: Home charging availability in 2022 is assumed to be close to the 2030 level in the reference scenario, and the growth rate of home charging availability before 2030 is much faster than in the reference scenario.
- Pessimistic scenario: Home charging availability in 2040 and 2050 are assumed to be 80% of 2040 and 2050 levels compared to the reference scenario. The levels of home charging availability in other years are linearly interpolated.

(g) Public charging infrastructure

Supplementary Figure 7 shows assumptions for public charging infrastructure: (a) Share of fast charging in public charging stations, (b) fast-charging power (kW), and (c) public charging availability. The limited electric range of BEV models is a significant contributor to the BEV range anxiety perceived by EV drivers. Therefore, widespread use of fast charging can lower this anxiety [19]. According to the China Automotive Technology and Research Center, the average ratio of fast charging in public charging stations was approximately 41.9%, and the most common fast-charging power in public stations was 40 kW in 2019 [6]. China has an ambitious NEV market; it is probable that the share of fast charging in public charging facilities will steadily grow in the future. To study the impact of charging infrastructure, the following scenarios are developed:

- Reference scenario:
  - Average share of fast charging in public charging stations is assumed to linearly increase.
  - Average fast-charging power is assumed to increase based on the government planning in China and potential trends of fast-charging technology [20,21].
- Better charging infrastructure scenario:
  - Average share of fast charging in public charging stations is assumed to be 150% of the 2050 reference share.
  - An average fast-charging power of 115 kW is assumed to be realized in 2023 instead of 2030 for the reference scenario.
  - Power rate will reach 400 kW by 2040, ten years earlier than in the reference scenario.
- Less-developed charging infrastructure scenario:
  - Average share of fast charging in public charging stations is assumed to be 80% of the 2050 reference share.
  - Average fast-charging power in 2030 will be 80% of the charging power for the same year under the reference scenario.
  - Power rate will reach 250 kW in 2050 (the peak charge rate delivered by Tesla's V3 Superchargers [22]).

The availability of public charging stations indicates the driving time to the nearest public charging station, which ranges from 1% to 100%. Here, 100% means a trip time at about 4–5 minutes, or about the average time driving to the nearest gas station in some urbanized areas in the U.S. [19,23] The following are assumed for distance to the nearest public charging for the above scenarios.

- Reference scenario: The availability of public charging stations is assumed to linearly increase from the current level of 5% (about a 25-minute drive to the nearest charging station) to 30% (about a 5-minute drive to the nearest charging station).
- Better charging infrastructure scenario: The availability of public charging stations is assumed to be 150% over the 2050 reference value.
- Less-developed charging infrastructure scenario: The availability of public charging stations is assumed to be 80% over the 2050 reference value. The values in other years are all linearly interpolated.

Supplementary Note 3. Scenarios Studied

Table 1 in the main text lists the scenarios studied in the work.

1. EX-ICEV: Assumes industry will meet the CAFC standards by aggressively improving ICEV efficiency without increasing BEV market share. After 2030, the FC of ICEVs decreases by 1% each year. The desired ICEV FC to meet CAFC regulations is presented in Supplementary Note 5.
2. EX-50%BEV: Assumes the market share of BEVs will increase linearly to 50% by 2030. The average FC of ICEVs for each MY is calculated to meet the corresponding CAFC standard. After 2030, the BEV market share is maintained at 50%, and the FC of ICEVs decreases 1% each year. The desired ICEV FC to meet the CAFC regulation is presented in Supplementary Note 5.
3. DC-Reference: Reference scenario described in the NEOCC model meeting the Dual Credit policy.
4. PT-CAFC: Similar to the DC-Reference scenario, but it removes the requirements by the NEV credit rules, and the only policy constraint is from the CAFC credit rules.
5. PT-BEVFC: Similar to the DC-Reference scenario, but it considers the electricity consumption (EC) of NEVs as FC in the CAFC regulation. The gasoline-electricity equivalency factor (GEEF), described in Supplementary Note 6, is used to convert the EC of NEVs to gasoline equivalent FC.
6. PT-M1.0: Similar to the DC-Reference scenario, but it sets the fuel-efficient multiplier to 1.0 instead of 0.5. As the fuel-efficient multiplier is changed from 0.5 to 1.0, the fuel-efficient ICEVs are no longer superior, policy-wise, in terms of calculating CFAC credits. Therefore, the number of fuel-efficient ICEVs could be less in this scenario than in the DC-Reference scenario.
7. DC-HOP: High oil price scenario defined in Supplementary Note 2.
8. DC-LOP: Low oil price scenario defined in Supplementary Note 2.
9. DC-LBC: Low battery pack cost scenario defined in Supplementary Note 2.
10. DC-BCI: Better charging infrastructure scenario defined in Supplementary Note 2.
11. DC-Optimistic: A scenario combining high oil price, low battery pack cost, and better charging infrastructure.

Supplementary Note 4. Uncertainties of GHG emissions

It is important to understand the robustness of the model because the results are highly dependent on the assumptions. In this section, we analyze the uncertainties of the following 4 assumptions used in the NEOCC model: NEV quota, battery cost, oil price, and charging infrastructure. For each analysis, the reference Dual Credit scenario is used as the baseline. We rerun the model by replacing the baseline assumption with the corresponding "high" and "low" scenarios presented in Supplementary Note 3. The new outputs of the NEOCC model are then adopted by the Fleet-China model to quantify the GHG emissions. The GHG emissions of electricity generation is a crucial factor that determines the emissions of BEVs. Thus, we calculate the uncertainties caused by the electricity GHG intensity for each analysis. The 6 °C and 2 °C electricity mix scenarios, described in the "Methods" section, are adopted as the "HighEle" and "LowEle" GHG emissions of the electricity generation.

(a) NEV Quota

In the reference Dual Credit scenario, we assume that the NEV quota linearly increases from 18% in 2023 to 40% ("DC_NEV40") in 2030. For the NEV quota uncertainty analysis, we set the low and high NEV quotas to 30% ("DC_NEV30") and 50% ("DC_NEV50") by 2030, respectively. The NEV quotas after 2030 remain the same as that of 2030, which is consistent with the baseline approach. We also keep the number of NEV credits granted to each NEV the same as the baseline, meaning it will gradually decrease to 1.0 by 2030.

Supplementary Figure 8 shows the GHG emissions under three NEV quota scenarios. The shaded area represents the uncertainties due to the GHG intensity of the Chinese electric grid. The results show that varying NEV quota from 30% to 50% does not have significant influence on the GHG emissions. In fact, achieving a CAFC target of 3.7 L/100km (WLTC-based FC, adjusted by the sales and vehicle curb weight) by 2030 is a much more challenging task than meeting the NEV quota targets. The NEOCC model shows that the CAFC rules are the major driving force that promotes the NEV market share before 2030. Improving ICEV fuel economy and increasing BEV market share simultaneously are the most cost-effective way for policy compliance. Meanwhile, after the NEV quota targets are exceeded, the surplus NEV credits are used to compensate the CAFC deficits under all three scenarios. Higher NEV quota (50%) reduces the surplus NEV credits available for compensating the deficit of CAFC credits, thus the market shares of NEV and "low FC ICEV" are shown a slight increase only. This finding is consistent with our previous results published in 2018, which showed that it is easier for the automotive industry to meet the NEV quota than to meet the CAFC targets and NEV credits are often used to make up the CAFC deficits [3].

No doubt, setting the NEV quota at a higher value can increase the NEV market share to meet the requirements. However, this is not free because higher internal subsidies are needed to further promote the sales of NEV and "low-FC ICEVs", which reduces the industry profits. Under the reference Dual Credit scenario with 40% NEV quota, the industry profit is 30% lower than the 2019 level. After evaluating a series of the government policies published in recent years and discussing with the experts and policymakers in China, we believe 40% can work as a reasonable number for the 2030 NEV quota and used in the reference Dual Credit scenario.

The GHG emissions of LDPV are sensitive to the GHG intensity of the electricity, which is no surprise as BEVs account for a significant market share after 2030. Under the high electricity GHG intensity

scenario, we observed up to 3% increase in annual GHG emissions and nearly 2% increase in cumulative GHG emissions from 2020 to 2040.

(b) Battery cost

We use the “high” (“DC_HBC”) and “low” (“DC_LBC”) battery cost scenarios described above for the uncertainty analysis. Lower battery cost promotes the sales of NEVs. As a consequence, the average FC of ICEVs slightly increases. The peak annual GHG emissions increase by 1.2% in 2032. The annual GHG emissions drop faster after 2032, and are also lower after 2036 than they are under the reference scenario. The opposite trend is observed under the “High” battery cost scenario. “High” battery costs reduce the annual GHG emissions before 2040, but we expect this trend will reverse after 2040 as the grid becomes cleaner.

In short, we observe remarkable changes in GHG emissions under different battery costs, as shown in Supplementary Figure 9. Although, the difference is not that significant (about 1%) before 2036, this difference will increase with the growth of BEV market share after 2040. It is worth noting that reducing battery cost alone is not adequate to boost NEV sales achieving the Dual Credit requirements. Industry internal subsidies are still needed for MYs before 2034.

The GHG emissions are sensitive to the GHG intensity of the electricity, as shown in Supplementary Figure 9. Under the high electricity GHG intensity scenario, we observe a 3.6% increase of the annual GHG emissions and a 2% increase of the cumulative GHG emissions from 2020 to 2040. Under the low battery cost and low electricity GHG intensity scenario, the annual GHG emissions are 10% lower than the reference scenario in 2040.

(c) Oil Price

The impacts of oil price on GHG emissions are presented in the main text. Oil price is the factor that significantly affected the total GHG emissions, which directly influences the gasoline price and the cost of ownership for ICEV as a result. Under the low oil price scenario (“DC-LOP”), improving ICEV fuel economy provides fewer fuel cost savings for consumers. Consequently, as shown in Supplementary Figure 10, we observe higher market share of high FC ICEVs, which causes higher average FC of new ICEVs and higher GHG emissions. Furthermore, modeling data reveals an interesting, yet counter intuitive observation: low oil price increases NEV market share. This is because of NEV credit requirements and the multiplier (0.5) used for fuel-efficient ICEVs. In effect, meeting NEV credit requirements means lower market share of fuel-efficient ICEVs, this in turn would mean more NEVs are sold to compensate for high sales volume of less efficient ICEVs. Likewise, in order to meet CAFC requirements, higher average FC of ICEVs commands higher NEV sales volume, all of which have zero FC under CAFC guidelines. Under the “DC-LOP” scenario, cumulative GHG emissions from 2020-2040 increase by 1.0% compared with the reference Dual Credit scenario.

The very opposite is observed under the high oil price (“DC-HOP”) scenario, as shown in Supplementary Figure 10. Under this scenario, significantly lower annual total GHG emissions are observed in comparison to the reference Dual Credit scenario, peaking at 782 Mt (-2.1%) by 2032. By 2040, we observe an 8.5% reduction in annual GHG emissions and the cumulative GHG emissions drop 3% compared with the reference scenario.

The GHG emissions are sensitive to carbon intensity of the Chinese electric grid. Under the high electricity GHG intensity scenario, the annual GHG emissions increase monotonically and the cumulative

GHG emissions increase by 2.8% by 2040. Under the HOP and low electricity GHG emission scenario, the annual GHG emissions are 16% lower than the reference scenario in 2040.

(d) Charging Infrastructure.

Two scenarios are created to represent better charging infrastructure (“DC_BCI”) and less charging infrastructure (“DC_LCI”). The BCI scenario considers optimistic home, public, and fast charging availability and higher fast charging power described in Supplementary Note 2. As discussed in the main text, improved charging infrastructure availability alone does not have a significant impact on annual total GHG emissions before 2040, less than 1% at all model years. Although BCI enhances the consumer acceptance of BEVs, it falls short of meeting the Dual Credit policy without sales incentives before 2036, as shown in Supplementary Figure 16. It, however, can reduce the overall incentive required, and hence eases the task for automakers to meet the policy requirements. Under the BCI scenario, we observe higher (0.9%) peak GHG emissions because of the higher BEV market share and slightly deteriorated ICEV FC. However, we do observe a faster decrease in annual GHG emissions after 2033, and the annual GHG emissions drop below that of the reference scenario after 2037, as shown in Supplementary Figure 11.

The GHG emissions are sensitive to the GHG intensity of the electricity. Under the high electricity GHG intensity scenario, we observe 3.3% increase in annual GHG emissions and 2% increase in cumulative GHG emissions from 2020 to 2040. Under the LCI and low electricity GHG emission scenario, the annual GHG emissions are 10% lower than the reference scenario in 2040.

(e) Policy tweak

This section presents the uncertainties of GHG emissions due to the GHG intensity of the electric grid, as shown in Supplementary Figure 12. Cleaner electricity grid not only helps reduce peak GHG emissions, but also advances the peak time. Under the low grid carbon intensity scenario, PT-BEVFC can reduce annual GHG emissions by 17% in 2040 and achieve nearly 10% reduction in cumulative GHG emissions from 2020 to 2040.

Supplementary Note 5. The average FC of ICEVs needed to meet the CAFC standards under the two extreme scenarios

Supplementary Figure 13 shows the required ICEV FC under the two extreme scenarios from 2020 to 2040. For the EX-50%BEV scenario, a less-than-20% reduction in ICEV FC from the current level is needed to meet the 2025 CAFC target. A minor ICEV FC rebound is observed from 2026 to 2030 due to the aggressive BEV penetration that offsets the need for additional ICEV FC improvement. In the EX-ICEV scenario, very aggressive ICEV efficiency improvement is needed. The new ICEV FC needs to be reduced to 5.0 L/100 km and 4.0 L/100 km by 2025 and 2030, respectively. This requires quick adoption of advanced energy saving technologies such as high efficient internal combustion engines and vehicle hybridization. It should be noted that, while this target is very aggressive, it still can be achieved. For example, the Nissan Note e-POWER, a series hybrid vehicle with energy fully provided by a 1.2-liter three-cylinder engine, could achieve 2.7 L/100 km under the JC08 driving cycle. [5]

Supplementary Note 6. Gasoline-electricity equivalency factor

The U.S. CAFE uses the petroleum equivalency factor (PEF) to convert the electricity consumption to the gasoline equivalent FC. However, the PEF values in the U.S. are not appropriate to use for China because of the differences in the energy sources used to generate electricity in the two countries. The "fuel-content" factor used to develop PEF is a legacy factor representing the gasoline content in E85, which is irrelevant for BEVs. In this study, we develop a new conversion factor based on the life-cycle GHG emissions, the gasoline-electricity equivalency factor (GEEF). It converts electricity consumption to the gasoline equivalent FC based on the life-cycle GHG emissions, which is calculated for each MY using the following equation:

$$GEEF = \frac{GHG_{Gasoline}}{GHG_{Electricity}}$$

where, GEEF is the gasoline-electricity equivalency factor (kWh/Liter), $GHG_{Electricity}$ is the GHG intensity of electricity generation (gCO_2/kWh), and $GHG_{Gasoline}$ is the GHG intensity of gasoline (gCO_2/Liter). In addition, we use IEA's 4°C grid scenario to calculate $GHG_{Electricity}$. Supplementary Figure 14 shows the change in GEEF over time. GEEF increases over time mostly because $GHG_{Electricity}$ decreases as the grid becomes cleaner.

Supplementary References

1. Yang, Z. & Cui, H. *Comments on China's proposed 2021-2025 fuel consumption limits, evaluation methods, and targets for passenger cars*. (2019).

2. Ministry of Industry and Information Technology. *<Fuel Consumption Evaluation Methods and Targets for Passenger Cars> National Standards Preparation Instructions*. (2018).

3. Ou, S. *et al.* The dual-credit policy: Quantifying the policy impact on plug-in electric vehicle sales and industry profits in China. *Energy Policy* **121**, 597–610 (2018).

4. The Strategic Advisory Committee & SAE-China. *Technology Roadmap for Energy Saving and New Energy Vehicles*. (China Machine Press, 2016).

5. Nissan. Equipment and Performance List of Nissan e-Power. *Nissan Japan* (2019). Available at: https://www2.nissan.co.jp/SP/NOTE/VLP/PDF/note_specsheet.pdf. (Accessed: 18th December 2019)

6. CATARC. *Automotive Data Center Database of China Automotive Technology and Research Center*. (2020).

7. Ou, S. *et al.* The retailed gasoline price in China: Time-series analysis and future trend projection. *Energy* 116544 (2019). doi:https://doi.org/10.1016/j.energy.2019.116544

8. Nissan. List of Main Equipment for Nissan Notebook e-Power S(WED). *Nissan Japan* (2019). Available at: https://www3.nissan.co.jp/vehicles/new/note/simulation.html#configure/BABSh/A3EQ/version. (Accessed: 8th July 2020)

9. Nissan. List of Main Equipment for Nissan Serena 2WD. *Nissan Japan* (2019). Available at: https://www3.nissan.co.jp/vehicles/new/serena/specifications.html. (Accessed: 8th July 2019)

10. Nykvist, B., Sprei, F. & Nilsson, M. Assessing the progress toward lower priced long range battery electric vehicles. *Energy Policy* **124**, 144–155 (2019).

11. Kittner, N., Lill, F. & Kammen, D. M. Energy storage deployment and innovation for the clean energy transition. *Nat. Energy* **2**, 17125 (2017).

12. Henze, V. Battery Pack Prices Fall As Market Ramps Up With Market Average At $156/kWh In 2019. *Bloomberg New Energy Finance* (2019). Available at: https://about.bnef.com/blog/battery-pack-prices-fall-as-market-ramps-up-with-market-average-at-156-kwh-in-2019/#:~:text=Partnership-,Battery Pack Prices Fall As Market Ramps Up With,At %2524156%252FkWh In 2019&text=Shanghai and London%252C December 3,to %2524156%25. (Accessed: 7th July 2020)

13. Schmidt, O., Hawkes, A., Gambhir, A. & Staffell, I. The future cost of electrical energy storage based on experience rates. *Nat. Energy* **2**, 17110 (2017).

14. Lutsey, N. & Nicholas, M. *Update on electric vehicle costs in the United States through 2030*. (2019).

15. Stevenson, M. Lithium-ion battery packs now $209 per kWh, will fall to $100 by 2025: Bloomberg analysis. *GreenCarReports* (2017). Available at: https://www.greencarreports.com/news/1114245_lithium-ion-battery-packs-now-209-per-kwh-

will-fall-to-100-by-2025-bloomberg-analysis. (Accessed: 7th July 2020)

16. Curry, C. *Lithium-ion battery costs and market*. *Bloomberg New Energy Finance* **5**, (2017).

17. Green, W. H. *et al. Insights Into Future Mobility: A Report from the Mobility of the Future Study*. (2019).

18. Ou, S., Lin, Z., He, X. & Przesmitzki, S. Estimation of vehicle home parking availability in China and quantification of its potential impacts on plug-in electric vehicle ownership cost. *Transp. Policy* **68**, 107–117 (2018).

19. Ou, S., Lin, Z., He, X., Przesmitzki, S. & Bouchard, J. Modeling charging infrastructure impact on the electric vehicle market in China. *Transp. Res. Part D Transp. Environ.* **81**, 102248 (2020).

20. Nicholas, M. & Hall, D. *Lessons learned on early electric vehicle fast-charging deployments*. (2018).

21. Collin, R., Miao, Y., Yokochi, A., Enjeti, P. & von Jouanne, A. Advanced electric vehicle fast-charging technologies. *Energies* **12**, 1839 (2019).

22. Tesla. Supercharging. (2020). Available at: https://www.tesla.com/support/supercharging. (Accessed: 28th July 2020)

23. Melaina, M., Bremson, J. & Solo, K. Consumer Convenience and the Availability of Retail Stations as a Market Barrier for Alternative Fuel Vehicles: Preprint. in (2013).
