# Supplementary material for: Greenhouse gas consequences of the China dual credit policy
Source: Nat Commun. 2020 Oct 15;11:5212. doi: 10.1038/s41467-020-19036-w (PMC7566593; doi:10.1038/s41467-020-19036-w)
Supplement: Supplementary file 3 — Reporting Summary [file 41467_2020_19036_MOESM3_ESM.pdf]

nature research

Corresponding author(s): He Xin, Shiqi Ou, Yu Gan

Last updated by author(s): Aug 27, 2020

Reporting Summary

Nature Research wishes to improve the reproducibility of the work that we publish. This form provides structure for consistency and transparency in reporting. For further information on Nature Research policies, see Authors & Referees and the Editorial Policy Checklist .

Statistics

For all statistical analyses, confirm that the following items are present in the figure legend, table legend, main text, or Methods section.

n/a	Confirmed	
[x]	[ ]	The exact sample size (*n*) for each experimental group/condition, given as a discrete number and unit of measurement
[x]	[ ]	A statement on whether measurements were taken from distinct samples or whether the same sample was measured repeatedly
[x]	[ ]	The statistical test(s) used AND whether they are one- or two-sided<br>*Only common tests should be described solely by name; describe more complex techniques in the Methods section.*
[x]	[ ]	A description of all covariates tested
[x]	[ ]	A description of any assumptions or corrections, such as tests of normality and adjustment for multiple comparisons
[x]	[ ]	A full description of the statistical parameters including central tendency (e.g. means) or other basic estimates (e.g. regression coefficient) AND variation (e.g. standard deviation) or associated estimates of uncertainty (e.g. confidence intervals)
[x]	[ ]	For null hypothesis testing, the test statistic (e.g. *F*, *t*, *r*) with confidence intervals, effect sizes, degrees of freedom and *P* value noted<br>*Give P values as exact values whenever suitable.*
[x]	[ ]	For Bayesian analysis, information on the choice of priors and Markov chain Monte Carlo settings
[x]	[ ]	For hierarchical and complex designs, identification of the appropriate level for tests and full reporting of outcomes
[x]	[ ]	Estimates of effect sizes (e.g. Cohen's *d*, Pearson's *r*), indicating how they were calculated

Our web collection on statistics for biologists contains articles on many of the points above.

Software and code

Policy information about availability of computer code

Data collection	The present study does not involve the collection of new raw data.
Data analysis	Microsoft Office Professional Plus 2016

For manuscripts utilizing custom algorithms or software that are central to the research but not yet described in published literature, software must be made available to editors/reviewers. We strongly encourage code deposition in a community repository (e.g. GitHub). See the Nature Research guidelines for submitting code & software for further information.

Data

Policy information about availability of data

All manuscripts must include a data availability statement. This statement should provide the following information, where applicable:

- Accession codes, unique identifiers, or web links for publicly available datasets
- A list of figures that have associated raw data
- A description of any restrictions on data availability

All data regarding the parameters used in the NEOCC model and China Fleet Vehicle model and data sources are documented in Supplementary Information. More specifically, the data used for NEOCC model calibration is presented in Supplementary Table 3. All other data (gasoline prices, battery cost, charging infrastructure availability, home charging availability, public charging availability) used in the study are given in Supplementary Figures 4-7. The details of NEV rules adopted in the NEOCC model is presented in Supplementary Tables 1-2. The sales, stocks and vehicle miles traveled used in the Base Case of China Vehicle Fleet Model are presented in Supplementary Table 4. The source data underlying all figures in the main manuscript and Supplementary Information are provided as a source data file. The NEOCC model and China Fleet Vehicle model are both available upon request after the manuscript is published.

Field-specific reporting

Please select the one below that is the best fit for your research. If you are not sure, read the appropriate sections before making your selection.

☐ Life sciences ☐ Behavioural & social sciences ☒ Ecological, evolutionary & environmental sciences

For a reference copy of the document with all sections, see nature.com/documents/nr-reporting-summary-flat.pdf

Ecological, evolutionary & environmental sciences study design

All studies must disclose on these points even when the disclosure is negative.

Study description	In this study, the New Energy and Oil Consumption Credits (NEOCC) model and the China Vehicle Fleet (China-Fleet) model are integrated and adopted for the GHG emission analysis. Both consumer choices, auto industry choices and Chinese government regulations are considered in order to quantify the GHG emissions as well as energy demand of the Chinese passenger vehicle fleet. Combining the NEOCC and China-Fleet models provides a pathway to assess the impact of policies and the external environment on transportation energy use and GHG emissions.
Research sample	The present study does not involve the collection of new raw data and data sampling. The data used in this work are documented in the main manuscript and Supplementary Information.
Sampling strategy	The present study does not involve the collection of new raw data and data sampling. The study projects the annual energy demand and annual GHG emissions.
Data collection	The present study does not involve the collection of new raw data. All data are from public resources, such as published papers, government statistical year books, government policy documents, etc. Vehicle market information for 2016-2019 were provided by China Automotive Technology and Research Center.
Timing and spatial scale	The present study does not involve the collection of new raw data.
Data exclusions	The present study does not involve the collection of new raw data and data exclusions.
Reproducibility	The present study does not involve experiments.
Randomization	The present study does not involve allocation of samples and randomization control.
Blinding	The present study does not involve data acquisition and blinding. This is not an experimental research, and does not need to create a control group for comparison.

Did the study involve field work? ☐ Yes ☒ No

Reporting for specific materials, systems and methods

We require information from authors about some types of materials, experimental systems and methods used in many studies. Here, indicate whether each material, system or method listed is relevant to your study. If you are not sure if a list item applies to your research, read the appropriate section before selecting a response.

Materials & experimental systems

n/a	Involved in the study	
☒	☐	Antibodies
☒	☐	Eukaryotic cell lines
☒	☐	Palaeontology
☒	☐	Animals and other organisms
☒	☐	Human research participants
☒	☐	Clinical data

Methods

n/a	Involved in the study	
☒	☐	ChIP-seq
☒	☐	Flow cytometry
☒	☐	MRI-based neuroimaging
